# A case report of Peutz–Jeghers syndrome in a child with Crohn's disease

**DOI:** 10.1002/jpr3.70184

**Published:** 2026-04-09

**Authors:** Hasala Rannulu, Eve May, Bethany Cunningham, Eric Cochran, Rachel Zdenek, Stefany H. Garrity

**Affiliations:** ^1^ Penn State College of Medicine Hershey Pennsylvania USA; ^2^ Penn State Department of Pediatrics Penn State Hershey Medical Center Hershey Pennsylvania USA; ^3^ Penn State Department of Pathology Penn State Hershey Medical Center Hershey Pennsylvania USA; ^4^ Penn State Department of Pediatric Genetics Penn State Hershey Medical Center Hershey Pennsylvania USA

**Keywords:** cancer surveillance in hereditary gastrointestinal syndromes, hamartomatous gastrointestinal polyposis, pediatric inflammatory bowel disease overlap

## Abstract

Peutz–Jeghers syndrome (PJS) is a rare genetic disorder characterized by hamartomatous polyps and mucocutaneous hyperpigmented freckles, whereas Crohn's disease (CD) is a condition characterized by chronic intestinal inflammation. Here, we present a rare case report of an 11‐year‐old male who presented with both CD and PJS. To our knowledge, this is only the fifth case report describing both of these diseases in the same patient. While CD is relatively common in industrialized countries, having CD in conjunction with PJS is exceedingly rare. This paucity of data describing the coexistence of the two conditions in the same patient has led to a general lack of guidance and screening recommendations in patients with both of these diseases. It is known that each disorder alone increases a patient's risk of malignancy; however, more data are needed to determine the appropriate cancer screening algorithm for patients with these dual diagnoses as well as management approaches.

## 1 INTRODUCTION

Peutz–Jeghers syndrome (PJS) is a rare condition caused by a mutation in the *STK11* gene, which serves as a possible tumor suppressor gene. This mutation in the *STK11* gene results in gastrointestinal polyposis, skin hyperpigmentation, and a greatly increased risk of malignancies of the gastrointestinal tract, lung, breast, uterus, and ovaries.^1^ The initial presentation of PJS can vary widely, but this disease frequently presents in childhood or adolescence with symptoms that can include sequelae of bowel obstruction or intussusception from hamartomatous gastrointestinal polyps, such as anemia, abdominal pain, and hematochezia.

Crohn's disease (CD) is a type of inflammatory bowel disease that is thought to result from a complex interplay between patients' genetics, environmental factors, and altered gut microbiota, ultimately leading to a dysregulated immune system. Patients with CD often present with diarrhea, abdominal pain, anemia, weight loss, and failure to grow.^2^

As such, the overlapping initial presentations of PJS and CD make it challenging for clinicians to identify and subsequently manage each condition, as CD and PJS differ in their long‐term implications, surveillance needs, and therapeutic goals. While CD management focuses primarily on controlling chronic inflammation and preventing complications such as strictures or growth failure, PJS management prioritizes cancer surveillance and endoscopic or surgical removal of hamartomatous polyps to prevent obstruction, bleeding, and malignant transformation. Given that dual diagnoses in an individual patient are exceedingly rare and poorly described in the literature, there is a critical gap in evidence‐based guidance on how to structure surveillance, sequence interventions, and coordinate multidisciplinary care.

## 2 CASE REPORT

A previously healthy 11‐year‐old boy presented to the pediatric surgery clinic with findings of a perianal abscess in the setting of abdominal pain, 8 lb weight loss, and diarrhea. Initial laboratory evaluation demonstrated elevated white blood cell count of 14.23 K/μL (normal < 13.5 K/μL) as well as an elevated C‐reactive protein of 0.57 mg/dL (normal < 0.5 mg/dL) and an erythrocyte sedimentation rate of 51 mm/h (normal < 10 mm/h) in the setting of normal hemoglobin and albumin levels. Subsequently, the patient went into the operating room for a rectal exam, incision and drainage of the perianal abscess, seton placement, proctoscopy, and rigid sigmoidoscopy under anesthesia.

**Figure 1:**
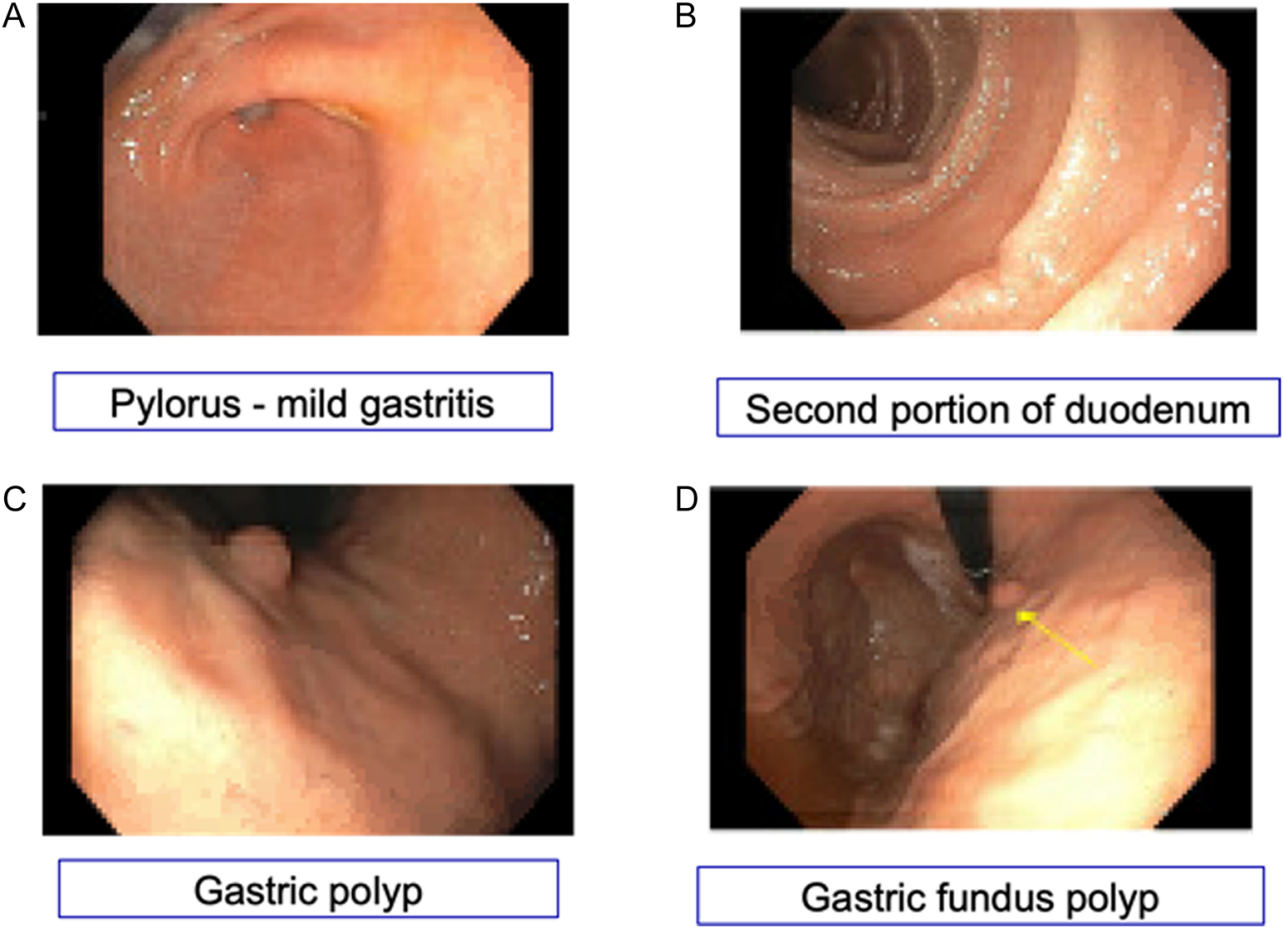
Upper endoscopy images. Panel (A) shows mild gastritis within the pylorus, (B) shows the second portion of the duodenum. Panel (C) shows a gastric polyp within the fundus, and (D) shows another gastric polyp at the fundus.

Further imaging via magnetic resonance enterography (MRE) demonstrated perianal fistula without abscess and small bowel wall thickening in the terminal ileum without penetrating disease or upstream dilation. His upper endoscopy was notable for mild gastritis with two gastric polyps in the fundus (Figure 1). These were removed via cold snare polypectomy. Pathologic analysis revealed them to be hyperplastic polyps. His colonoscopy was grossly normal. The pathology results from his upper endoscopy and colonoscopy were notable for subacute and chronic inflammation with granulomas present in his terminal ileum and throughout his colon. He was diagnosed with perianal and ileocolonic CD (Paris A1b L3 B3p G0) and started on infliximab 10 mg/kg as well as ciprofloxacin and metronidazole for his perianal disease. At his outpatient gastrointestinal follow‐up visit, he was noted to have distinct freckles on both his upper and lower lip as well as peri‐oral mucosa and had a genetics referral placed for a concern of PJS, which confirmed the presence of the *STK11* mutation.

**Figure 2:**
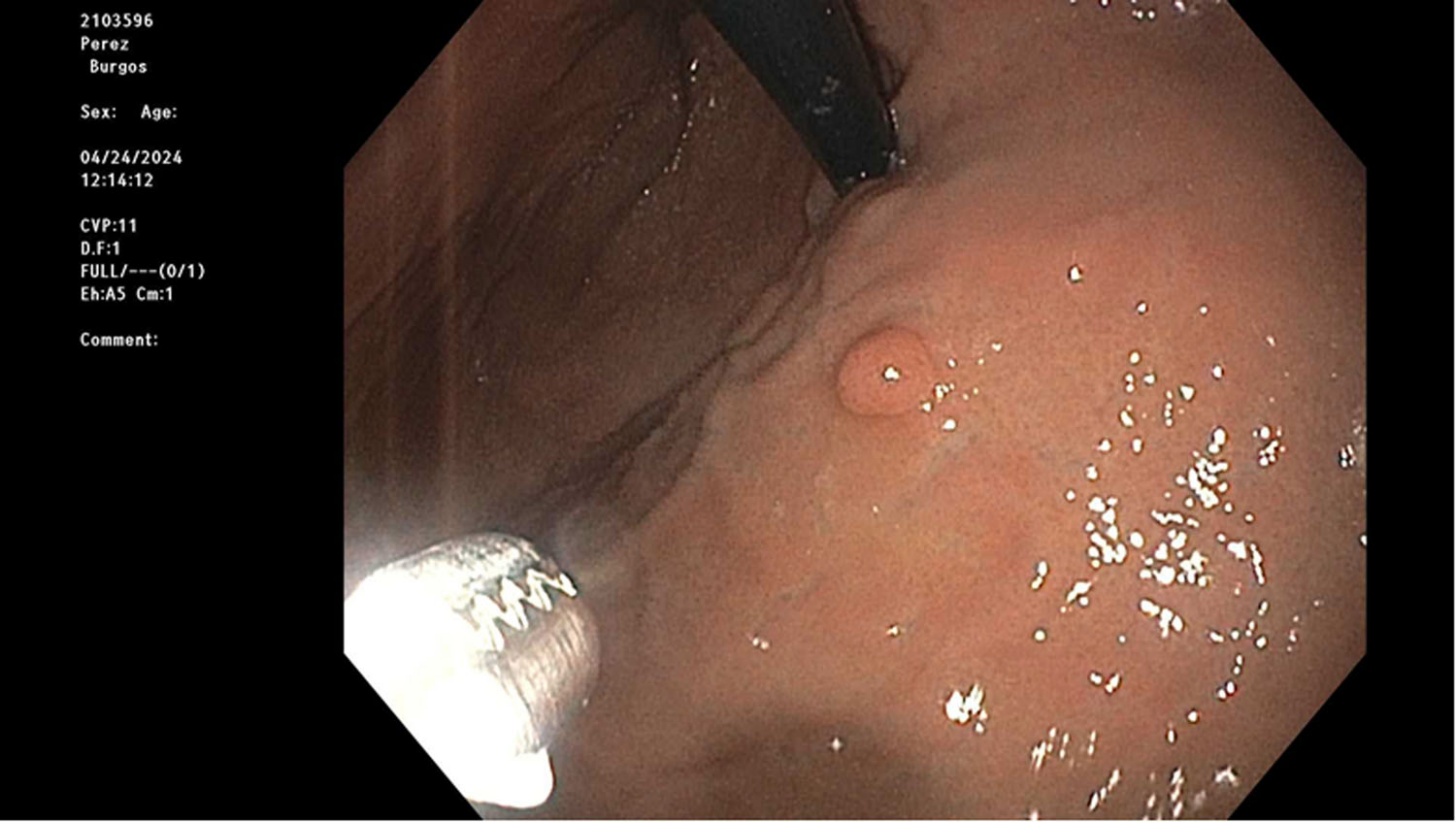
Gross image of gastric polyp.

**Figure 3:**
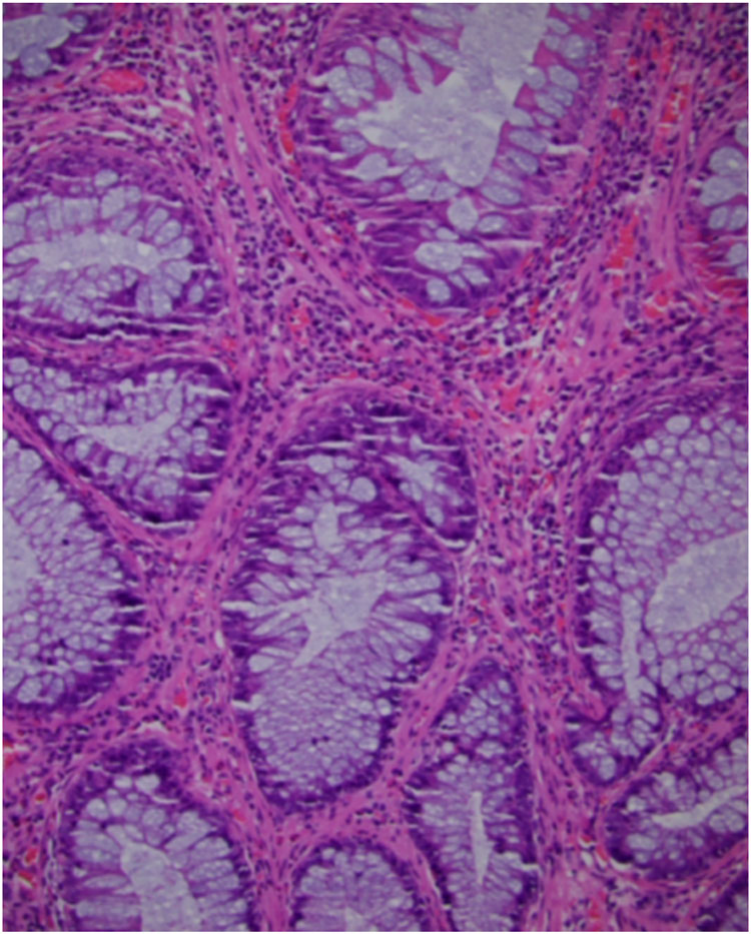
Histological image of gastric polyp characterized by thin arborizing pattern of elongated smooth muscle in between the crypts, consistent with PJS. PJS, Peutz‐Jeghers syndrome.

One year after his initial diagnosis, the patient had a repeat upper endoscopy and colonoscopy. This was notable for one small gastric polyp on gross examination in the fundus, which was biopsied (Figure 2). Pathological analysis of the gastric polyp demonstrated an arborizing pattern of thin, elongated smooth muscle between the crypts consistent with a typical PJS polyp (Figure 3). At the time of his last visit to our institution, he was on dual therapy with infliximab 12.5 mg/kg every 4 weeks plus methotrexate with an infliximab trough of 14 μg/mL. Although he had improved from the initial presentation, he continued to have fatigue, abdominal pain, and rare diarrhea. His erythrocyte sedimentation rate remained elevated despite the above efforts, and he was also being treated for Vitamin D deficiency and low ferritin levels. Over the course of 12 months, his body mass index improved from 22.8 to 24.4 as he gained 22 pounds. At this point in his disease course, the patient moved out of state and was lost to follow‐up at our institution.

## 3 DISCUSSION

While inflammatory bowel diseases like CD are relatively common in industrialized countries, having CD in conjunction with PJS is exceedingly rare. To our knowledge, there are only four other published reports of CD arising in patients with PJS: a 1984 case in Germany,^3^ a 2013 case in South Korea,^4^ a 2019 case in the United Kingdom,^5^ and a 2024 case in Morocco.^6^

Current guidelines for surveillance for PJS, according to the American College of Gastroenterology (ACG), recommend lifelong, multidisciplinary surveillance for cancers of the small bowel, colon, stomach, pancreas, breast, ovary, uterus, cervix, and testes.^7^ As such, the ACG recommends small bowel surveillance every 2–3 years using video capsule endoscopy (VCE) or MRE starting in childhood or adolescence and upper endoscopy/colonoscopy at similar intervals. For CD, the ACG recommends that patients with Crohn's colitis involving more than 30% of the colon for at least 8 years undergo regular colonoscopic surveillance for colorectal cancer, but does not recommend routine small bowel cancer surveillance in CD alone.^8^

The authors of this paper suggest all organ‐specific surveillance recommended for PJS should be maintained, and colonoscopic surveillance for CD‐associated dysplasia should be coordinated with PJS protocols, typically resulting in colonoscopies at least every 2–3 years. In a patient with both conditions, surveillance for PJS will often exceed the intensity and scope of standard CD surveillance, particularly regarding small bowel evaluation. The need for regular small bowel imaging in PJS can incidentally provide additional information about CD activity or complications, potentially allowing for earlier detection of strictures, inflammation, or neoplasia than would be achieved by CD surveillance alone. Conversely, colonoscopic surveillance for CD‐associated dysplasia can be coordinated with PJS polyp surveillance, reducing procedural burden and improving adherence.

Related to this, polypectomy in patients with both PJS and CD also remains a point of contention. Standard of care in PJS involves the removal of polyps to prevent later complications.^7^ This is not always the case in CD, where risks of polypectomy, such as bleeding or perforation, can be magnified, especially in the setting of active inflammation.^8^ The AGA recommends polypectomy for PJS polyps >0.5 cm in the stomach and colorectum and >1–1.5 cm in the small bowel, but in CD, these interventions should be individualized and performed with heightened caution.^7^, ^8^ Given the complexity of managing PJS and CD, a personalized approach integrating regular surveillance, medical management, and timely endoscopic or surgical interventions when necessary is essential to optimizing patient outcomes and minimizing risks.

## 4 CONCLUSION

This case highlights the lack of data describing the coexistence of the PJS and CD in pediatric patients, as both conditions can present with overlapping gastrointestinal symptoms—including abdominal pain, bleeding, anemia, and weight loss—which can make differentiation challenging, particularly at the time of initial presentation. Standard disease‐specific guidelines are insufficient in isolation. Instead, coordinated care across gastroenterology, genetics, oncology, surgery, and radiology is essential to address the unique challenges posed by dual pathology. At this time, further research—including registry development, longitudinal outcome studies, and expert consensus—is critically needed to inform evidence‐based surveillance algorithms, refine timing and modality of interventions, and optimize long‐term outcomes for this vulnerable patient population. Until such data is available, clinicians must maintain a broad differential, adopt proactive surveillance strategies, and apply meticulous procedural planning to mitigate complications and improve the quality of life for patients with concurrent PJS and CD.

## CONFLICT OF INTEREST STATEMENT

The authors declare no conflicts of interest.

## ETHICS STATEMENT

This manuscript was written with the express written informed consent of the patient's mother. All patient information has been deidentified.
